# Cost-effectiveness of polymyxin B hemoperfusion for septic shock: an observational study using a Japanese nationwide administrative database

**DOI:** 10.1186/s44158-023-00087-6

**Published:** 2023-02-15

**Authors:** Kenji Fujimori, Kunio Tarasawa, Kiyohide Fushimi

**Affiliations:** 1 Department of Health Administration and Policy, Tohoku University Graduate School of Medicine, Sendai, Japan; 2 Department of Health Policy and Informatics, Tokyo Medical and Dental University Graduate School of Medical and Dental Sciences, Bunkyo-Ku, Tokyo, Japan

**Keywords:** Polymyxin B hemoperfusion, PMX, Sepsis, DPC database, Cost-effectiveness, Propensity score matching

## Abstract

**Background:**

Polymyxin B hemoperfusion (PMX) removes endotoxin from septic shock patients. Although the treatment has been clinically used for more than 20 years, its cost-effectiveness has not been evaluated in detail.

**Methods:**

This study used the Japanese diagnosis procedure combination (DPC) administrative database from April 2018 to March 2021. We selected adult patients with a primary diagnosis of sepsis and the SOFA score at the sepsis diagnosis was between 7 and 12. The patients were divided into the PMX group that received PMX treatment and the control group that did not receive PMX. After adjusting the patient background by propensity score matching, we calculated the incremental cost-effectiveness ratio (ICER) by determining the difference in quality-adjusted life-year (QALY) and medical cost between the PMX and the control groups.

**Results:**

Nineteen thousand two hundred eighty-three patients were included in the study. Among them, 1492 patients received PMX treatment, and 17,791 did not. As a result of 1:3 propensity score matching, 965 patients in the PMX group and 2895 patients in the control group were selected and analyzed. Twenty-eight-day mortality and hospital mortality were significantly lower in the PMX group. The average medical cost per patient of the PMX group was 31,418 ± 21,144 Euro and that of the control group was 24,483 ± 21,762 Euro, with a difference of 6935 Euro. Life expectancy, life year-gained (LYG), and the QALY were 1.70, 0.86, and 0.60 years longer in the PMX group, respectively. The ICER was calculated to be 11,592 Euro/year, which was lower than the reported willingness-to-pay threshold of 38,462 Euro/year.

**Conclusion:**

Polymyxin B hemoperfusion was shown to be an acceptable treatment in terms of the medical economy.

## Background

Sepsis is a life-threatening organ dysfunction caused by a dysregulated host response to infection [1]. It is the leading cause of death in the ICU, and the mortality rate is very high when it progresses to septic shock [2].

The treatment of septic shock includes early administration of antimicrobial agents, infusion of fluids, and administration of vasopressors. When these standard treatments are not successful, one of the option treatments is blood purification therapy to remove toxins and inflammatory mediators from the patient’s circulating blood. Direct hemoperfusion using polymyxin B-immobilized fibers, polymyxin B hemoperfusion (PMX), is a treatment targeting endotoxin. This bacterial component triggers whole-body inflammation and causes organ dysfunctions [3, 4].

Numerous studies on PMX have shown its clinical effectiveness in improving hemodynamics and pulmonary functions of septic shock patients [5, 6]. On the other hand, results of several randomized controlled studies (RCT) on PMX evaluating its survival benefit are controversial [7, 8]. In observational studies using an extensive Japanese database, we have reported that PMX is effective in reducing mortality and the number of days on organ support [9, 10]. In particular, we have found that the effectiveness of PMX is higher in patients with a moderate degree of organ dysfunction with sequential organ failure assessment (SOFA) scores in the range of 7–12 [11].

PMX is a relatively expensive treatment compared to the standard therapies for septic shock, such as antimicrobials, fluid infusions, and vasopressors. In the Surviving Sepsis Campaign (SSC) guideline 2021 [12], PMX treatment is rated as “suggest against” (weakly recommended not to be used). One of the reasons for this recommendation is the high cost of the treatment. However, there has been little information on the cost-effectiveness of PMX treatment. Therefore, it is an important issue to evaluate the validity of the cost of this treatment to the therapeutic effect.

In this study, we examined the cost-effectiveness of PMX treatment using a sizable Japanese inpatient database, the Diagnosis Procedure Combination (DPC).

## Methods

### Data source and patients

This retrospective observational study used inpatient data included in the Japanese DPC database. The DPC database contains discharge and administrative claims data for all inpatients discharged from more than 1000 participating hospitals, covering 92% of all tertiary-care emergency hospitals in Japan [13, 14].

We extracted the patient data from April 2018 to March 2021. Selected patients were aged 20 years or older and whose primary diagnosis was sepsis based on the International Classification of Diseases 10th Revision (ICD-10) codes. Since we found in our previous study that PMX is effective on the patients whose baseline SOFA scores are 7–12, we targeted these patients in this analysis. Therefore, we excluded the patients whose SOFA score is below seven or above 12. We also excluded the patients who died within 3 days after the diagnosis of sepsis, were hospitalized for more than 125 days, transferred to other hospitals within 28 days without recovery, or received their first PMX treatment other than on the first or second day of sepsis diagnosis.

We defined the first SOFA score record as the day of sepsis diagnosis. We categorized patients who received PMX on the first or second day of sepsis diagnosis into the PMX group and patients who did not receive PMX into the control group.

### Propensity score matching

We performed a propensity score matching analysis between patients with PMX-treated (PMX group) and non-treated (control group). We estimated the propensity score using a logistic regression model for the use of PMX as a function of the following confounders: age, gender, emergency versus elective hospital admission, university hospital versus non-university hospitals, admission to the emergency room (ER) or intensive care unit (ICU), Charlson Comorbidity Index (CCI), continuous renal replacement therapy (CRRT), hemodialysis (HD), mechanical ventilation, surgery, administration of γ-globulin, AT III, rTM, steroid, red blood cell transfusion, platelet transfusion, and the maximum daily dose of noradrenaline. A one-to-three matched analysis using the nearest-neighbor matching was performed based on the estimated propensity score of each patient. We used a caliper width of 0.2 of the standard deviation of the propensity score. We evaluated the balance among covariates using absolute standardized difference (ASD), which considers a difference below 10% to be well balanced.

### Clinical effects

For the cohort after propensity score matching, we compared the following patient outcomes between the PMX group and control group; 28-day-mortality, mortality at hospital discharge, length of hospital stay, ventilator-, vasopressor-, and CRRT-free days at day 28. Patients who were discharged alive and who died in the hospital were included in assessing the length of hospital stay. Free days were counted as zero when a patient died before day 28.

### Cost

We obtained the medical costs for each patient from the day of sepsis diagnosis to the day of discharge from the DPC data. We received the costs separately in the following categories; hospital visit fees and management fees, prescription, injection, treatment, surgery/anesthesia, laboratory test, diagnostic imaging, rehabilitation, general hospitalization fees, ER/ICU hospitalization fees, and meals. The difference in total medical costs between the PMX group and the control groups was used to evaluate cost-effectiveness.

All costs were obtained in Japanese yen (JPY) and converted to Euro. The conversion to Euro was calculated at 130 JPY per Euro as the conversion rate as of March 2022.

### Evaluation of cost-effectiveness ratio

The expected life expectancy after discharge was calculated using each patient’s age and the Japanese life expectancy table. Patients who survived sepsis have a higher risk of death. A reduction rate of 0.51 for the life expectancy has been reported for the adjustment of this risk [15, 16]. We used this value to calculate the life-year gained (LYG) by multiplying the life expectancy by 0.51 as the following formula.

$$LYG=life\;expectancy\;after\;discharge\times0.51$$

In addition, we multiplied LYG by a utility weight of 0.69 to estimate quality-adjusted life-year (QALY), as the formula below, considering the lower quality of life (QOL) after hospital discharge in sepsis survivors [15, 16].

$$QALY = LYG \times 0.69$$

The incremental cost-effectiveness ratio (ICER), which is the difference in cost divided by the difference in expected life expectancy, is the most commonly used measure for determining the cost-effectiveness. We calculated the ICER using the following formula

$$ICER=(the\;average\;cost\;of\;the\;PMX\;group-the\;average\;cost\;of\;the\;control\;group)/(\mathrm{the}\;\mathrm{average}\;\mathrm{QALY}\;\mathrm{in}\;\mathrm{the}\;\mathrm{PMX}\;\mathrm{group}-\mathrm{the}\;\mathrm{average}\;\mathrm{QALY}\;\mathrm{in}\;\mathrm{the}\;\mathrm{control}\;\mathrm{group})$$

### Sensitivity analysis

We obtained the adjustment mentioned above factors for calculating LYG and QALY (0.51 and 0.69) from the old literature published before 2010, and the values are likely to be inaccurate. In light of this uncertainty, we examined the variation of ICERs when we changed the adjustment factors. In one case, ICERs were calculated using reduced rates of 0.3 for the LYG calculation and 0.6 for the QALY calculation. In the other case, we used reduced rates of 0.7 for the LYG calculation and 0.8 for the QALY calculation. Finally, we compared the results with the value obtained under the standard condition (base case).

### Subgroup analysis

As a subgroup analysis, we examined the impact of severity of organ damage and the cause of sepsis on cost-effectiveness. First, we stratified the patients by SOFA score range into two groups, 7–9 and 10–12. We also divided patients into two groups by the cause of sepsis, those with abdominal infection and those with other sites of infection, and calculated ICERs for each combination. Patients with abdominal infections were identified primarily by using the ICD-10 code descriptions.

### Statistical analysis

We reported continuous variables as mean and standard deviation (SD) and categorical variables as number and percentage. We performed statistical analysis using JMP Pro 15.2.0 (SAS Institute Inc.) We used the *χ*^2^ test (Pearson method for *p* value) to compare two groups for mortality and the Wilcoxon rank-sum test to compare the length of hospital stay and the free days.

## Results

### Patients

**Fig. 1:**
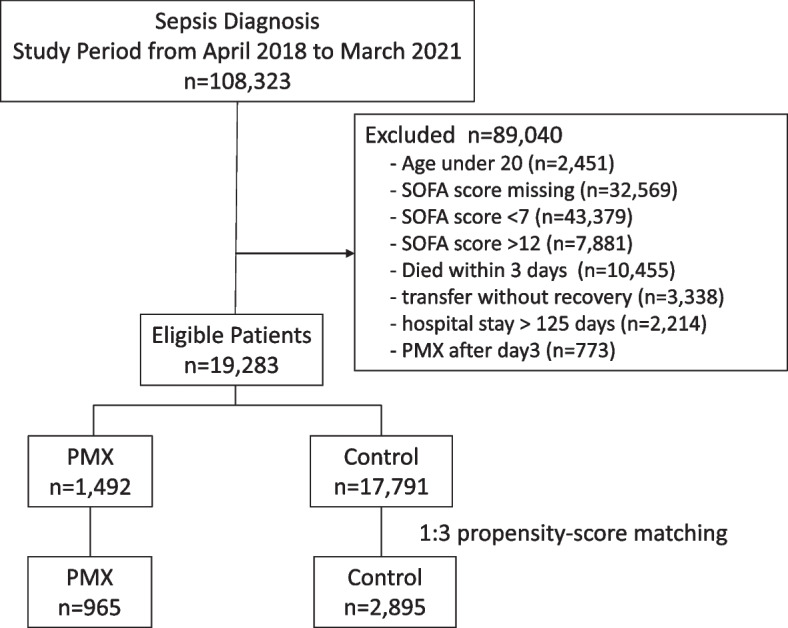
Patient selection flow

During the study period, 108,323 patients were admitted with a primary diagnosis of sepsis. Among them, 19,283 patients were included in the study after excluding 89,040 patients who were younger than 20 years old, patients with SOFA scores less than seven or higher than 12, patients with missing SOFA score data, patients who died within three days after sepsis diagnosis, patients hospitalized for more than 125 days, patients transferred to other facilities without recovery, or patients treated with PMX after day 3. One thousand four hundred ninety-two patients received PMX treatment among the eligible patients, and 17,791 did not. The number of shock patients, defined as cardiovascular SOFA score of 2 or higher, was 1144/1492 (76.7%) for PMX-treated patients and 9573/17,791 (53.8%) for untreated patients. As a result of 1:3 propensity score matching, 965 patients in the PMX group and 2895 patients in the control group were selected (Fig. 1).

Table 1 shows the patient baseline before and after propensity score matching. Before the matching, the PMX group had a younger age, a higher proportion of emergency and ER/ICU admissions, and a higher proportion of university hospitals than the control group. The PMX group also had higher CRRT use, ventilator use, surgery rate, and higher rates of γ-globulin, rTM, AT III, steroid administration, and red blood cell and platelet transfusions. The maximum daily dose of noradrenaline was also higher in the PMX group. After propensity score matching, the ASD was less than 10% for all factors, confirming that this study balanced elements between the groups well.

**Table 1:** Baseline patient characteristics before and after propensity score matching

	Unmatched			Matched		
	PMX (*n* = 1492)	Control (*n* = 17,791)	ASD%	PMX (*n* = 965)	Control (*n* = 2895)	ASD%
Age, mean (SD)	72.7 (12.8)	77.2 (12.7)	35.1	72.9 (12.5)	73.9 (13.4)	0.2
Sex (male), *n* (%)	847 (46.8)	9792 (55.0)	13.6	548 (56.8)	1635 (56.5)	0.5
Emergency admission, *n* (%)	1336 (89.5)	17,196 (96.7)	25.3	883 (91.5)	2662 (92.0)	1.3
University hospital, *n* (%)	331 (22.2)	2130 (12.0)	23.3	186 (19.3)	565 (19.5)	0.5
ER/ICU admission, *n* (%)	1094 (73.3)	8237 (46.3)	45.9	670 (69.4)	2037 (70.4)	1.7
CCI, mean (SD)	1.9 (1.7)	1.9 (1.7)	1.6	1.9 (1.7)	2.0 (1.8)	1.3
CRRT, *n* (%)	950 (63.7)	1914 (10.8)	115.0	491 (50.9)	1418 (50.0)	1.5
HD, *n* (%)	71 (4.8)	734 (4.1)	2.5	59 (6.1)	160 (5.5)	2.0
Mechanical ventilation, *n* (%)	962 (64.5)	3901 (21.9)	79.6	528 (54.7)	1576 (54.4)	0.5
Surgery, *n* (%)	817 (57.8)	2443 (13.7)	89.8	418 (43.3)	1278 (44.2)	1.4
γ-globulin, *n* (%)	492 (33.9)	1313 (7.4)	60.5	242 (25.1)	672 (23.2)	3.6
rTM, *n* (%)	744 (49.9)	1604 (9.0)	89.8	356 (36.9)	960 (33.2)	6.4
AT III, *n* (%)	430 (28.8)	843 (4.7)	62.7	199 (20.6)	528 (18.2)	5.0
Steroid, *n* (%)	700 (46.9)	4614 (25.9)	37.3	428 (44.4)	1245 (43.0)	2.2
RBC transfusion, *n* (%)	572 (38.8)	2155 (12.1)	55.3	306 (31.7)	854 (29.5)	3.9
Platelet transfusion, *n* (%)	240 (16.1)	832 (4.7)	34.1	124 (12.9)	350 (12.1)	1.9
Maximum noradrenaline, mean (SD)	14.3 (12.9)	7.4 (9.9)	60.2	12.9 (11.6)	12.8 (13.1)	0.6

*ASD* Absolute standard difference, *SD* Standard deviation, *ER* Emergency room, *ICU* Intensive care unit, *CCI* Charlson Comorbidity Index, *CRRT* Continuous renal replacement therapy, *HD* Hemodialysis, *rTM* recombinant thrombomodulin, *AT* Antithrombin, *RBC* Red blood cell

### Clinical effects

Table 2 shows the comparison of the clinical outcomes between the PMX group and the control group. The 28-day mortality rate was 16.8% in the PMX group and 23.9% in the control group (*p* < 0.0001). And the hospital discharge mortality rate was 24.4% in the PMX group and 34.1% in the control group (*p* < 0.0001), significantly lower in the PMX group. Ventilator-free, vasopressor-free, and CRRT-free days at day 28 were significantly longer in the PMX group, showing better recovery of organ dysfunctions. For the length of hospital stay, the PMX group was substantially longer than the control group (median 32 days vs. 25 days). This finding probably reflects that more people in the control group died in the hospital in a short time.

**Table 2:** Comparison of the clinical outcomes in the matched cohort

	PMX (*n* = 965)	Control (*n* = 2895)	*P* value
28-day mortality, *n* (%)	162 (16.8)	692 (23.9)	< 0.0001
Hospital mortality, *n* (%)	235 (24.4)	987 (34.1)	< 0.0001
Length of hospital stay, days, median (IQR)	32 (18–51)	25 (15–45)	< 0.0001
Ventilator free days, days, median (IQR)	19 (0–24)	15 (0–23)	< 0.0001
Vasopressor free days, days, median (IQR)	25 (20–26)	24 (0–26)	< 0.0001
CRRT free days (day), days, median (IQR)	23 (13–25)	20 (0–24)	< 0.0001

*IQR* Interquartile range, *CRRT* Continuous renal replacement therapy

### Cost-effectiveness ratio

Table 3 shows the calculation results of the average medical costs from the day of sepsis diagnosis to the day of discharge. The total medical cost per patient of the PMX group was 31,418 ± 21,144 Euro and that of the control group was 24,483 ± 21,762 Euro. A difference of 6935 Euro indicates that the PMX group had higher medical costs. Of the various cost categories, the largest difference was in the “treatment” cost, probably because it includes the cost of PMX treatment. “Injections,” “aurgery/anesthesia,” “diagnostic imaging,” “rehabilitation,” “general hospitalization fee,” and “meals” were also more expensive in the PMX group. This result may reflect that the average length of hospital stay was longer in the PMX group than the control group.

**Table 3:** Cost and clinical effects

	PMX (*n* = 965)	Control (*n* = 2895)	Difference	*P* value
Hospital visit/management (SD)	142 (119)	126 (118)	16	0.0003
Prescription (SD)	250 (497)	247 (614)	3	0.8693
Injection (SD)	5677 (8175)	4827 (6552)	850	< 0.0001
Treatment (SD)	7514 (4564)	3053 (4821)	4461	< 0.0001
Surgery/anesthesia (SD)	4321 (7032)	3724 (8208)	597	0.0261
Laboratory test (SD)	1045 (1268)	1004 (1482)	40	0.3644
Diagnostic imaging (SD)	749 (579)	663 (625)	86	< 0.0001
Rehabilitation (SD)	726 (873)	57 (819)	190	< 0.0001
General hospitalization fee (SD)	4493 (3374)	4013 (3560)	480	< 0.0001
ER/ICU hospitalization fee (SD)	6093 (4564)	5947 (4726)	146	0.3995
Meals (SD)	408 (357)	341 (362)	67	< 0.0001
**Total cost (SD)**	**31,418 (21,144)**	**24,483 (21,762)**	**6935**	** < 0.0001**
Life expectancy (years)	12.93	11.23	1.70	
LYG (years)	6.59	5.73	0.86	
QALY (years)	4.55	3.95	0.60	
**ICER (Euro/year)**			**11,592**	

Costs are expressed as mean (SD) in Euro

*SD* Standard deviation, *LYG* Life year gained, *QALY* Quality-adjusted life-year, *ICER* Incremental cost- effectiveness ratio

Table 3 also shows comparisons of life expectancy, LYG, and QALY. Life expectancy was 12.93 years in the PMX group and 11.23 years in the control group, 1.70 years longer in the PMX group. LYG, calculated by multiplying life expectancy by 0.51, was 0.86 years longer in the PMX group. QALY was calculated by multiplying LYG by 0.69 and was 0.60 years longer in the PMX group. The ICER calculated from the differences in cost and in QALY was 11,592 Euro/year (1,507,019 JPY/year).

### Sensitivity analysis

We performed a sensitivity analysis to examine the effects of changing the adjustment factors used to estimate LYG and QALY from the life expectancy years. The results are shown in Table 4. The ICER for the combination of 0.3 for the reduction rate and 0.6 for utility weight was 22,663 Euro/year. The ICER for the combination of 0.7 and 0.8 was estimated to be 7285 Euro/year.

**Table 4:** Sensitivity analyses

	Reduction rate for LYG estimation	Utility weight for QALY estimation	Difference in cost (Euro)	Difference in life expectancy (yrs)	Difference in LYG (years)	Difference in QALY (years)	ICER (Euro/year)
Case 1 (base case)	0.51	0.69	6935	1.70	0.87	0.60	11,592
Case 2	0.3	0.6	6935	1.70	0.51	0.31	22,663
Case 3	0.7	0.8	6935	1.70	1.19	0.95	7285

*LYG* Life year gained, *QALY* Quality-adjusted life-year, *ICER* Incremental cost-effectiveness ratio

### Subgroup analysis

Table 5 shows the results of the subgroup analysis combining SOFA scores stratified into the 7–9 and 10–12 ranges and the site of infection divided into abdomen and others. The lowest ICER was observed in the patients with abdominal infections with SOFA 10–12 (4,102 Euro/year). Conversely, the highest ICER was observed in patients with non-abdominal infections with SOFA 7–9 (13,263 Euro/year).

**Table 5:** Subgroup analyses

SOFA range	Cause of sepsis	The number of patients (*n*)		Hospital mortality (%)		Total cost (Euro)		Difference in cost (Euro)	Difference in life expectancy (yrs)	Difference of LYG (years)	Difference of QALY (years)	ICER (Euro/year)
		PMX	Control	PMX	Control	PMX	Control					
7–9	Abdominal	153	274	15.7	34.3	31,734	25,782	5952	3.40	1.73	1.20	4974
7–9	Others	333	1190	24.0	28.7	28,374	21,373	7001	1.50	0.77	0.53	13,263
10–12	Abdominal	153	305	24.8	44.6	35,723	31,883	3840	2.66	1.36	0.94	4102
10–12	Others	326	1126	28.5	37.0	32,359	25,448	6911	1.58	0.81	0.56	12,429

*SOFA* Sequential organ failure assessment, *LYG* Life year gained, *QALY* Quality-adjusted life-year, *ICER* Incremental cost-effectiveness ratio

## Discussion

This study evaluated the cost-effectiveness of PMX treatment by using the Japanese nationwide inpatient database, the DPC data. We compared the propensity score-matched cohort in the SOFA score range of 7–12. We found the QALY was longer in the PMX group by 0.60 years. The cost was higher in the PMX group by 6935 Euro. The ICER was calculated to be 11,592 Euro/year (1,507,019 JPY/year).

Willingness-to-pay threshold (WTP) is an estimate of what a consumer of health care is prepared to pay for the health benefit. Reportedly, the willingness-to-pay threshold for QALY in Japan is 38,462 Euro/QALY (5 million JPY/QALY) [17–19]. Compared to this value, the ICER of PMX treatment is sufficiently low, indicating that it is an acceptable treatment in terms of the medical economy.

Close examination of the cost difference between patients with and without PMX treatment shows that the increase in the cost of the “treatment” is the largest at 4461 Euros, reflecting the cost of PMX treatment. Other costs, such as “injection” and “hospitalization” were also slightly higher in the PMX group. This result may reflect a longer hospital stay in the PMX group reflecting the lower in-hospital mortality.

Sepsis survivors have shorter life expectancy after being discharged than healthy people. To adjust for this fact, we calculated LYG by multiplying the life expectancy by a factor of 0.51, based on a previous study. In addition, 0.69 was used as the utility weight when calculating QALY from LYG. However, these reduction rates are derived from old studies, and the values are likely to be inaccurate. Therefore, we calculated ICERs for cases with smaller values (0.3 and 0.6) and larger values (0.7 and 0.8) for both reduction factors as a sensitivity analysis to see the impact of these uncertainties. As a result, we calculated ICERs to be 7285 Euro and 22,663 Euro, respectively, showing that the ICER is below the acceptable WTP threshold of ICER (38,462 Euro/QALY) even in the worst case.

In a subgroup analysis, we stratified patients by the SOFA score (range 7–9 vs. 10–12) and the cause of sepsis (into abdominal infection vs. others). For SOFA score, ICER values were lower for 10–12 than 7–9. For the site of infection, ICERs were lower for abdominal infection than for others. However, the ICER values for all subgroups were within the acceptable WTP threshold.

One previous study on the cost-effectiveness of PMX reported an incremental cost per LYG of 3864 Euros [20]. However, this study analyzed data from one randomized controlled trial conducted in Italy (the EUPHAS study). This study limited the number of analyzed patients to 64. In addition, the study was conducted in the years 2004–2007, about 15 years ago. We assume the overall treatment of sepsis may have been different in some respects from what it is today. Our study used new real-world data obtained from 2018 to 2021, and the number of patients analyzed was approximately 4000 cases. Therefore, we believe our results are more accurate and reflect actual clinical practice in Japan.

There are several limitations to this study. First, although we performed propensity score matching, we cannot rule out the existence of unadjusted confounders not included in the DPC data. Second, the disease code of sepsis is based on clinical judgement and not base on the SEPSIS-3 definition. Third, blood test values, including blood cell counts, biomarkers, and endotoxin levels, were not available from the DPC data. Fourth, survivors’ medical conditions, treatment costs, and life expectancy vary from country to country. Fifth, the adjustment factors used for the calculation of LYG and QALY were derived from the old literature and may not be precise. With this in mind, we performed a sensitivity analysis with varying adjustment factors. Finally, the results of this study are an evaluation of cost-effectiveness using data from Japan. Therefore, readers may not extrapolate them directly to other countries.

## Conclusions

Our study evaluated the cost-effectiveness of polymyxin B hemoperfusion treatment for sepsis using a Japanese nationwide administrative database. The treatment’s incremental cost-effectiveness ratio (ICER) was calculated as 11,592 Euro/QALY using propensity score-matched cohorts. This cost was sufficiently low compared to the reported willingness-to-pay threshold. However, due to limitations as an observational study, the clinical efficacy of polymyxin B hemoperfusion and its relation to medical costs should be confirmed in future studies.

## Data Availability

The datasets used and/or analyzed during the current study are available from the corresponding author on reasonable request.

## Abbreviations

ASD: Absolute standard difference
AT: Antithrombin
CCI: Charlson comorbidity index
CRRT: Continuous renal replacement therapy
DPC: Diagnosis procedure combination
ER: Emergency room
HD: Hemodialysis
ICER: Incremental cost-effectiveness ratio
ICU: Intensive care unit
IQR: Interquartile range
JPY: Japanese yen
LYG: Life-year gained
PMX: Polymyxin B hemoperfusion
QALY: Quality-adjusted life-year
RBC: Red blood cell
RCT: Randomized controlled trial
rTM: Recombinant thrombomodulin
SD: Standard deviation
SOFA: Sequential organ failure assessment
WTP: Willingness to pay
